# Toxic comments are associated with reduced activity of volunteer editors on Wikipedia

**DOI:** 10.1093/pnasnexus/pgad385

**Published:** 2023-12-05

**Authors:** Ivan Smirnov, Camelia Oprea, Markus Strohmaier

**Affiliations:** Graduate Research School, University of Technology Sydney, 15 Broadway, Sydney 2007, Australia; Department of Computer Science, RWTH Aachen University, Ahornstrasse 55, Aachen 52074, Germany; Business School, University of Mannheim, L 15 1–6, Mannheim 68161, Germany; GESIS—Leibniz Institute for the Social Sciences, Unter Sachsenhausen 6–8, Köln 50667, Germany; Complexity Science Hub Vienna, Josefstaedter Strasse 39, Vienna 1080, Austria

## Abstract

Wikipedia is one of the most successful collaborative projects in history. It is the largest encyclopedia ever created, with millions of users worldwide relying on it as the first source of information as well as for fact-checking and in-depth research. As Wikipedia relies solely on the efforts of its volunteer editors, its success might be particularly affected by toxic speech. In this paper, we analyze all 57 million comments made on user talk pages of 8.5 million editors across the six most active language editions of Wikipedia to study the potential impact of toxicity on editors’ behavior. We find that toxic comments are consistently associated with reduced activity of editors, equivalent to 0.5–2 active days per user in the short term. This translates to multiple human-years of lost productivity, considering the number of active contributors to Wikipedia. The effects of toxic comments are potentially even greater in the long term, as they are associated with a significantly increased risk of editors leaving the project altogether. Using an agent-based model, we demonstrate that toxicity attacks on Wikipedia have the potential to impede the progress of the entire project. Our results underscore the importance of mitigating toxic speech on collaborative platforms such as Wikipedia to ensure their continued success.

**Significance Statement**

While the prevalence of toxic speech online is well studied, its true impact on the productivity of online communities remains largely unexplored. In this study, we focus on Wikipedia, which as the largest and most-read online reference, serves as a vital source of knowledge for millions of users worldwide. By analyzing all comments made over 20 years on user talk pages of 8.5 million editors across multiple language editions, we demonstrate that toxic speech is associated with a significant loss in the productivity of Wikipedia editors. These findings may have broad implications for large-scale collaborative projects and online communities, emphasizing the need to promote healthy and sustainable communication practices to protect crucial online information ecosystems and ensure their long-term success.

## Introduction

Wikipedia is arguably one of the most successful collaborative projects in history. It has become the largest and most-read reference work ever created, and it is currently the fifth most popular website on the Internet (1). Millions of users worldwide rely on Wikipedia as their first source of information when encountering a new topic, for fact-checking and in-depth research (2). Even if caution might be required when consulting less actively maintained pages (3), numerous studies have shown that Wikipedia is a reliable source of information in areas ranging from political science (4) to pharmacology (5) and its accuracy is comparable to traditional encyclopedias (6) and textbooks (7).

One of the most remarkable aspects of Wikipedia’s success is that its content is exclusively created and curated by volunteer editors, known as Wikipedians. The English edition alone has more than 120,000 active editors (8). However, this volunteer-driven model also makes Wikipedia susceptible to the inherent challenges associated with maintaining such a large online community (9, 10). For example, it has been previously observed that Wikipedia is not free of conflict, particularly in the form of so-called edit wars (11), which impose significant costs on the project (12) and could negatively affect the quality of Wikipedia articles (13).

In this paper, we focus on the impact of toxic comments directed toward editors on their activity. This aspect is less studied, but potentially not less important, as affected by toxic comments, Wikipedians might reduce their contributions or abandon the project altogether, threatening the success of the platform (14).

Toxicity has been extensively studied on popular social media websites such as Twitter (15, 16), Reddit (17, 18), and similar platforms (19, 20). However, much of these research focuses on automated toxicity detection and prevalence estimation rather than on evaluating its impact (21). As an online encyclopedia, Wikipedia is often perceived as immune to toxicity and has a strict “No personal attacks” policy (22). Despite that, toxic speech and harassment have been previously observed on the platform (23–27). The effects of such behaviors on editors’ contributions are, however, not well understood nor well studied. The largest study to date relies on a voluntary opt-in survey of the 3,845 Wikipedians conducted in 2015 (24). It reports that 20% of users witnessing harassment have stopped contributing for a while, 17% considered not contributing anymore and 5% stopped contributing at all.

In this paper, we analyzed all 57 million comments made on user talk pages of editors on the six most active language editions of Wikipedia (English, German, French, Spanish, Italian, Russian) to understand the potential impact of toxic speech on editors’ contributions (see Methods and materials section for our definition of toxic comments). User talk pages are a place for editors to communicate with each other either on more personal topics or to extend their discussion from an article’s talk page. The majority of toxic comments are left on user talk pages (28). The comments we study were extracted from revision histories of talk pages and, thus, include even those toxic comments that were later archived or deleted by the page owner.

**Fig. 1.**
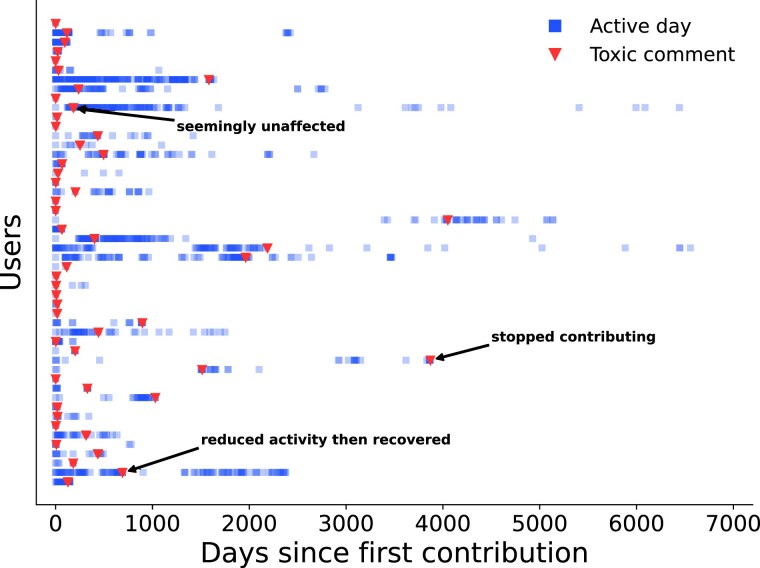
After receiving a toxic comment many users temporarily reduce their activity or leave the project completely. The figure shows the activity of 50 randomly selected users who received exactly one toxic comment. Blue squares indicate an active day, i.e. a day when at least one edit was done, starting from the first contribution of a given user. Red triangles correspond to toxic comments. Note that while some users are resilient and their activity is seemingly unaffected by toxic comments, many users temporarily reduce their activity or stop contributing altogether.

Figure 1 shows the activity of 50 randomly selected users who have received exactly one toxic comment. While some users are seemingly unaffected by a toxic comment, others temporarily reduce their activity or leave the project completely. The aim of our paper is to quantify this effect on the entire population of editors.

We estimate the number of lost active days associated with a toxic comment by comparing the number of active days before and after receiving a toxic comment. To account for potential baseline change, we have matched editors that received a toxic comment with similarly active editors who received a nontoxic comment. We have separately studied if toxic comments increase the probability of editors leaving the project altogether. Finally, we have used an agent-based model to model the potential impact of an increased number of toxic comments on Wikipedia.

## Results

### Loss of editor activity

**Fig. 2.**
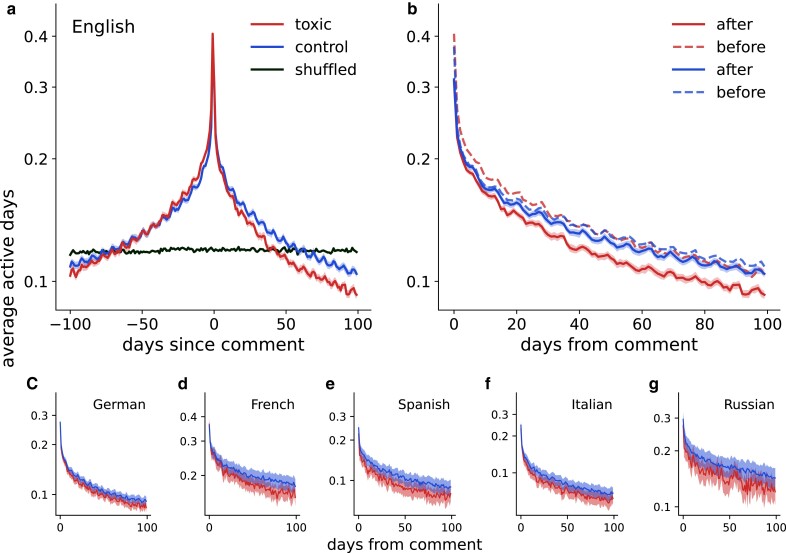
After receiving a toxic comment, users become less active. On average, users are more active near the time when they receive a toxic comment (peak at zero for the red line in panel a). Average activity across all users who have received a toxic comment is lower in all 100 days after the event compared to the corresponding days before (dashed and solid red lines in panel b). This cannot be explained by a baseline drop in activity after a nontoxic comment (dashed and solid blue lines in panel b). Similar results hold not only for the English edition but also for the other five editions (c–g).

To estimate the potential effect of a toxic comment, we compute the proportion of users who were active on day X before or after receiving a toxic comment (Fig. 2). We find that, on average, editors are more active near the time when they receive a toxic comment, with a peak at 24 h prior to the comment. At this time point, more than 40% of editors were active, as shown by the red line in Fig. 2a. This is a rather unsurprising observation since toxic comments are often made as a reaction to an edit made by a user and, thus, users are expected to be active around the time of a toxic comment. Note that if the timestamps around which the curve is centered are shuffled (black line in Fig. 2a) then this pattern disappears completely as expected.

We also find that average activity across all users who have received a toxic comment is lower during all 100 days after the event compared to the corresponding days before (dashed and solid red lines in Fig. 2b), e.g. smaller number of users is active five days after receiving a toxic comment than five days before receiving it. To rule out the possibility that this is due to a general drop in activity over time or a drop in activity after any comment, we select a control group of users who have received a nontoxic comment, and whose average activity in the 100 days before the comment is the same as the average activity of users who received a toxic comment (see Methods and materials section for details).

We observe a similar characteristic peak around the nontoxic comment, likely due to both toxic and nontoxic comments being reactions to a contribution made by an editor. However, in contrast to a toxic comment, a nontoxic comment does not lead to a significant decrease in activity (dashed and solid blue lines in Fig. 2b). Similar results hold for all six language editions that we have examined (Fig. 2c–g).

We then estimate the lost activity associated with a toxic comment by computing the decrease in activity after a toxic comment, taking into account a potential baseline drop, i.e. by computing $\Delta =({\mathrm{After}}_{\mathrm{toxic}}-{\mathrm{Before}}_{\mathrm{toxic}})-({\mathrm{After}}_{\mathrm{nontoxic}}-{\mathrm{Before}}_{\mathrm{nontoxic}})$. We find that this loss is statistically significant for all language editions studied (Table 1). We further explored the robustness of this result with respect to the toxicity threshold and potential filtering of users according to their activity. As expected, for higher toxicity thresholds, i.e. for more severely toxic comments, the effect is stronger (Supplementary Fig. S1). Considering only active users also leads to higher estimates; however, here we are reporting a conservative estimate, i.e. no filtering is used for results presented in Fig. 2 and Table 1.

**Table 1.** Lost active days in the 100 days following a toxic comment.

Edition	*Δ*	*P*-value	$N_{users}$
English	−1.207	$2.6\times 10^{-66}$	36,332
German	−0.546	$1.5\times 10^{-7}$	10,346
French	−1.851	$4.8\times 10^{-9}$	2,239
Spanish	−0.563	$8.6\times 10^{-3}$	2,446
Italian	−0.336	$2.3\times 10^{-2}$	3,567
Russian	−1.219	$7.8\times 10^{-4}$	1,134

The lost active days are estimated by computing the difference between the number of active days during 100 days after a toxic comment and the number of active days during 100 days before a toxic comment. This difference is then compared with the baseline drop after a nontoxic comment, i.e. $\Delta =({\mathrm{After}}_{\mathrm{toxic}}-{\mathrm{Before}}_{\mathrm{toxic}})-({\mathrm{After}}_{\mathrm{nontoxic}}-{\mathrm{Before}}_{\mathrm{nontoxic}})$. The *P*-value is computed using Student’s *t*-test.

While these results demonstrate that our findings are not limited to one language, they should not be used to compare effects between language editions, as there is no guarantee that the same toxicity threshold for the toxicity detection algorithm will have the same meaning in different languages.

Note that given that thousands of users have received at least one toxic comment (Supplementary Table S1), even a moderate loss per user could result in many human-years of lost productivity for Wikipedia in the short run. By multiplying the estimated loss per user from Table 1 by the number of users who have received at least one toxic comment, we could estimate the total loss of activity that is ranging from 5 human-years for Russian Wikipedia to 265 human-years for the English edition. The reason for the lasting effect of toxicity is that some new users might be discouraged by a toxic comment and choose to leave the project altogether after just a few contributions. This means that a single toxic comment could deprive Wikipedia of a potentially long-term contributor.

To further investigate this effect, we compare the probability of leaving Wikipedia after receiving a toxic comment with the probability of leaving Wikipedia after receiving a nontoxic comment.

### Leaving Wikipedia

**Fig. 3.**
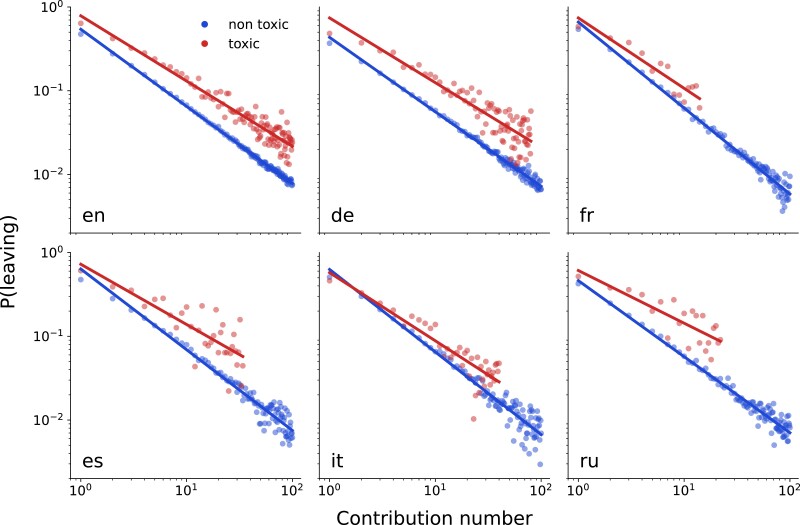
The probability of leaving Wikipedia after receiving a toxic comment is substantially higher than might be expected otherwise. For all six editions the probability of leaving declines with the number of contributions. At the same time, this probability is substantially higher after receiving a toxic comment than might be expected otherwise. Dots are probability estimates and solid lines are the best linear fit on a log-log scale.

We observed that the probability of leaving Wikipedia after *N* contributions declines with *N*. $P_{N}(\text{leaving})$ is approximately proportionate to $N^{-\alpha }$, where α ranges from 0.89 to 1.02, indicating a long-tailed distribution. While the probability of leaving the project after the first and only contribution is high ($P_{1}=47\%$ for English Wikipedia), the risk of leaving Wikipedia drops to 0.7% for users who have made 100 contributions. To study the potential effects of toxic comments, we separately consider contributions that are followed by a toxic comment and contributions that are not followed by a toxic comment (see Methods and materials section for details). We find that the risk of an editor leaving after a toxic comment is consistently higher for all editions and regardless of the contribution number, see Fig. 3. We provide an analysis of the significance of these findings in Supplementary Fig. S4.

### Agent-based modeling

As has been demonstrated above, toxic comments increase the likelihood of editors abandoning Wikipedia. If enough editors leave, this could potentially impede the progress of the project as a whole. In order to estimate the potential impact of toxic comments, we model users’ behaviors by varying the toxicity of the environment, ranging from a nontoxic environment, where the probability of a user leaving follows the empirically observed nontoxic probability distribution, $P_{N}^{\mathrm{non}}$ (blue dots in Fig. 3), to a highly toxic environment, where the probability of leaving corresponds to an empirically observed toxic probability distribution, $P_{N}^{\mathrm{tox}}$ (red dots in Fig. 3). We also consider a potential attack targeted at new users. In this scenario, each user receives a toxic comment after their first and second contributions, e.g. their probability of leaving after the first and second contribution is defined by $P_{N}^{\mathrm{tox}}$, and after that follows the empirically observed $P_{N}$.

For our modeling, we focus on a cohort of users who made their first contribution between the 4,000th and 6,000th day from the first recorded contribution to English Wikipedia in our dataset. We opted for this timeframe as it reflects Wikipedia’s current phase characterized by a relatively consistent number of active editors. This period follows the site’s initial exponential growth and a subsequent decline but comes before the anomalous increase in activity due to the COVID-19 pandemic (see Discussion section for details on these stages).

For our modeling, we employed an agent-based approach. Each day, agents (representing users) join Wikipedia and make their first contribution. The number of agents joining each day is equal to the actual count of first-time contributors to English Wikipedia on that particular day. After their first contribution, agents keep contributing, following a Poisson process, i.e. in such a way that the distance between two consecutive contributions, *D*, follows an exponential distribution: D∼Exp(λ), where *λ* is estimated from empirical data. After each contribution, the agent’s probability of leaving the project is determined by the toxicity level, *T*, and the empirically observed distributions $P_{N}^{\mathrm{non}}$ and $P_{N}^{\mathrm{tox}}$. In particular, after *N*’s contribution the user leaves the project with probability $T*P_{N}^{\mathrm{tox}}+(1-T)*P_{N}^{\mathrm{non}}$. If the toxicity level is 0, then the probability of leaving follows the nontoxic distribution $P_{N}^{\mathrm{tox}}$, and if the toxicity level is 1, then the probability of leaving follows the toxic distribution $P_{N}^{\mathrm{tox}}$.

After the initial 2,000 days, no new agents join the project; however, we continue to model the behavior of the remaining agents for the subsequent 2,000 days, for which we have available empirical data for comparison.

**Fig. 4.**
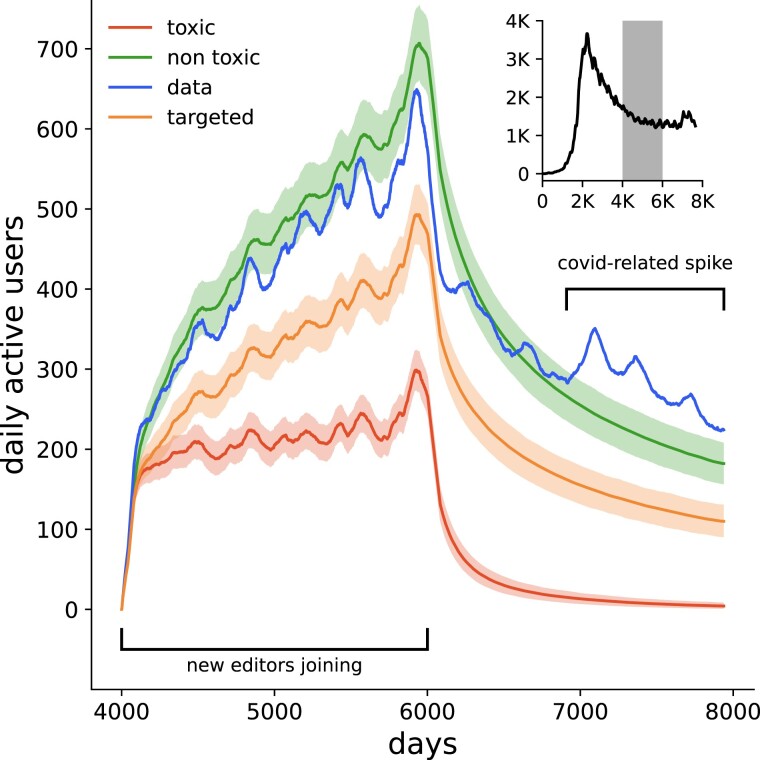
High levels of toxicity and targeted attacks could significantly reduce the number of active editors. Modeling results for a cohort of editors making their first contribution during the relatively stable phase of Wikipedia (shaded region in the inset). The model reproduces the general dynamics of user activity (blue line) but, as expected, cannot capture the COVID-19-related spike in activity. An extreme level of toxicity (red line) could reduce the cohort to virtually no active users, contrasting with a nontoxic environment (green line) or actual activity (blue line). Targeted attacks on newcomers (orange line) have the potential to significantly reduce the number of active contributors.

Our model generally reproduces the dynamics of user activity (Fig. 4), though, as expected, it cannot account for a later COVID-19-induced spike in activity. We find that an extreme level of toxicity could effectively reduce the cohort to almost no users in the long run, compared to the sustained numbers in a nontoxic setting or as observed in the data. Additionally, targeted attacks on newcomers have the potential to significantly decrease the number of active users, posing a risk to the project. The detailed results of our modeling, showing the effects of different toxicity levels on user count, are presented in Supplementary Fig. S6.

## Discussion

We conducted a large-scale analysis, covering all comments made on user talk pages of the six most active language editions of Wikipedia over a period of 20 years, and found that toxic comments are associated with a decreased activity of editors who have received these comments and an increased risk of them leaving the project altogether. Additionally, via agent-based modeling, we showed that toxicity attacks on Wikipedia have the potential to impede the progress of the entire project.

The main limitation of our study is its relatively narrow scope, as it focuses solely on the association between toxic comments left on user talk pages and the subsequent decrease in users’ activity. However, this approach allowed us to formulate our findings with precision and ensure their robustness. We believe that our study complements and extends existing studies on Wikipedia and online communities more broadly, and may serve as a foundation for further exploration of the effects of toxicity, as we discuss in this section.

###  

#### Conflict on Wikipedia

Conflict on Wikipedia has already been a subject of numerous studies, with particular attention given to so-called “edit wars” (11, 29, 30). These arise when groups of editors, disagreeing about page content, repeatedly override each other’s contributions. It has been estimated that edit wars can impose substantial conflict and coordination costs on Wikipedia (12). Furthermore, it has been demonstrated that these costs increase over time and a smaller proportion of the total work by Wikipedians directly contributes to new article content. Conflict could also undermine content quality. For instance, the level of conflict on discussion pages, as assessed by raters, has been shown to negatively correlate with the quality of the corresponding Wikipedia articles (13).

In contrast to previous studies, our focus is on comments left on user talk pages rather than article talk pages. While this narrows the scope of our study, it also ensures that the comments we examine are directly addressed to a specific editor. Our approach also mitigates potential bias that could be introduced by the topic of an article. For instance, comments on talk pages linked to articles about violence might be misclassified as toxic by an algorithm due to the presence of highly negative keywords.

It is possible that toxic comments we observe on user talk pages are not independent from a broader conflict occurring elsewhere on Wikipedia. Therefore, it is conceivable that the effect we observe is not purely explained by toxic comments, but also by a broader conflict which leads both to a toxic comment on a user talk page and decreased activity of this user. Future research is needed to address this limitation and explore the context in which toxic comments occur.

It is worth noting, however, that it has already been established that toxicity on its own could lead users to stop contributing either temporarily or permanently, as this is what editors themselves report in surveys (24). Our study complements such studies by providing an estimate of the potential effects while also being performed on a scale that is not achievable by survey methods.

#### Stages of Wikipedia life cycle

Wikipedia has not grown linearly but has instead passed through several stages. It began with exponential growth (31), which subsequently slowed (32). Following that, the number of active users declined before Wikipedia entered its current stage, characterized by a relatively stable number of active users (33), with a slow decline observed in some language editions. A notable exception was a temporary spike in activity due to the COVID-19 pandemic (34). See Supplementary Fig. S5 for an illustration of these patterns in the editions studied in this paper.

It has been found that the main reason for halted growth is a sharp decline in the retention of newcomers (35). Specifically, with the project’s development, the rejection of newcomer contributions has increased, demotivating them and driving them away. Our results complement these findings by highlighting that newcomers are also particularly vulnerable to toxic comments. If users receive a toxic comment after their first or second contributions, their chances of continuing to contribute are 1.8 times lower compared to users who did not receive toxic comments.

#### Diversity of editors

Wikipedia is often considered a neutral and unbiased source of knowledge. In fact, this is ingrained in its “Neutral point of view” policy, which is officially one of the five fundamental principles of Wikipedia (36). However, the claim of neutrality should not be accepted uncritically (37). For instance, while Wikipedia mandates that its content is supported by reliable sources, the selection of these sources can significantly deviate from the norms of the expert knowledge community, introducing biases to Wikipedia content (38). Even if the content of articles is neutral, their coverage may be biased. It is well documented, for example, that biographies of women are underrepresented on Wikipedia (39). Wikipedia’s own rules might contribute to such biases. For instance, providing reliable sources as required by Wikipedia for biographies of women might be challenging because fewer sources exist on women due to historic inequalities (40). Another case in point is the Oral Citations project, which aimed to use oral citations for content on countries that are underrepresented in other sources (41). However, this initiative was met with opposition by the English Wikipedia community.

These content biases are closely connected to the lack of diversity among editors (38, 42). While estimates vary, the vast majority of Wikipedians are men (43). Notably, Wikipedia did not achieve its own goal of having at least 25% women editors by 2015 (44). This shortfall is a significant concern for the project, as diversity can improve the quality of content and reduce its biases (13, 45). While multiple barriers confront women editors on Wikipedia (40, 46, 47), toxicity is likely to be one of key factors contributing to the observed gender imbalance. Specifically, research has shown that while men and women are equally likely to face online harassment and abuse, women experience more severe violations (48). They are also more likely to be affected by such incidents and to self-censor in an attempt to prevent potential harassment (48). This has been confirmed in the Wikipedia context as well, where it has been demonstrated that the psychological experiences of women and men editors differ, leading to higher attrition rates among women (49). Similar results were found in another survey (24), showing that women experiencing toxicity are more likely to stop contributing in the future.

Overall, there are reasons to believe that toxicity might significantly undermine the diversity of Wikipedia editors, which can, in turn, compromise the quality of Wikipedia articles and introduce biases in its coverage. This underscores the importance of our findings. While most of the existing studies focus on the gender gap, we want to emphasize that the Wikipedia diversity problem goes beyond that, including racial, nonbinary, and other biases as well (50–52). For instance, we observed that many of the toxic comments in our data set include ethnic slurs. Future studies are needed to better understand the experiences of minority groups on Wikipedia and the effects that toxicity has on them.

#### Interventions

The Wikipedia community is well aware of the aforementioned problems, and there have been multiple efforts to address them through various interventions. Research into reward systems showed that while they might work effectively for already highly productive editors, they fail to motivate less active editors (53). Another study found no significant effect of positive rewards in online communities (54).

To address the gender gap in Wikipedia content, numerous events dedicated to creating entries about women were organized (46). An analysis of such interventions, which focused on two popular feminist interventions, confirmed that they succeeded in introducing content about women that would otherwise be missing (55). However, there is still a need to address the gender gap on a more systematic and sustainable level. For instance, one study showed that most of the women activists who attended editing workshops later chose not to continue contributing to Wikipedia, citing safety concerns as their primary reason (46). This issue was echoed in another study which identified safety as a core concern for women editors (56).

A suggested solution to this problem has been the red-flagging of harassment and harassers (46). However, the opinion that toxic comments are negligible and should be seen as merely over-enthusiastic participation is still present among editors (25). Furthermore, various anti-harassment measures have been declined multiple times by the community, as they were seen to slow the process of content creation (57, 58). Based on our findings, we believe there is a need to reevaluate these policies, and more research attention is required to understand the impact of potential interventions.

#### The wider role of peer-production systems

Wikipedia plays a crucial role in the global information infrastructure, aiming to provide millions of people with access to free, unbiased knowledge. Due to its reputation as a neutral and comprehensive information source, it has become a trusted first choice source of knowledge for many and its articles frequently appear in top search engine results (59, 60). In fact, studies have shown that Google search results rely heavily on Wikipedia, and the quality of these results significantly diminishes without Wikipedia (61). Beyond search engines, Wikipedia was shown to be valuable to other online communities such as Stack Exchange and Reddit (62).

While Wikipedia is arguably the most successful peer-production system, it is certainly not the only one. Others include hundreds of wikis hosted by Fandom, the numerous question-and-answer communities of Stack Exchange, and various other platforms ranging from online maps to online learning (33). Interestingly, for these projects, the same patterns that are typical of Wikipedia have been observed (63), i.e. the initial growth in number of contributors is followed by a decline characterized by a decreased retention of newcomers. This suggests that our findings might have broader implications for large-scale collaborative projects and online communities. It emphasizes the need to promote healthy and sustainable communication practices to protect crucial online information ecosystems and ensure their long-term success.

## Methods and materials

### Data and preprocessing

#### Comments on user talk pages

The Wikimedia Foundation provides publicly accessible dumps of all the different wikis’ content.^a^ These dumps are updated on a regular basis, with complete revision history dumps generated once per month. For this paper, we used the English dump from 2021 November 1, the German dump from 2022 August 1, the French, Italian, and Spanish dumps from 2022 August 1, and the Russian dump from 2022 July 1. The data was obtained from a mirror hosted by the Umeå University, Sweden.^b^

From the dumps, the user talk pages were extracted. A user’s talk page is a place where other editors can communicate with the user either on more personal topics or to extend their discussion from an article talk page. When the comments left on the talk page are resolved or become too old, users can choose to archive them. This helps them keep better track of new incoming topics. Once archived, the old comments are not displayed on the talk page anymore but are rather linked in a subpage. Nevertheless, the entire history of the user talk page, as of any other page on Wikipedia, can be fully seen under the tab of revision history. The revision history records one entry for every edit made on the page saving each time the complete content of the page. Thus retrieving a single comment requires performing the difference between two consecutive revisions. The Wikimedia API does offer a method to compute the difference between two revisions, however, applying it on a scale that was necessary for this research was unfeasible. For that reason, we developed our own parser to extract comments as a difference between two versions of the page (64).

We excluded from our analysis talk pages that belong to unregistered users, e.g. users who are represented only by an IP address rather than a user name, because IP addresses are dynamic and it can not be assumed that one address represents a single user throughout Wikipedia history. Additionally, we have excluded comments made by officially registered bots. Comments that were made by users on their own pages are also not considered.

When extracting comments, we cleared wiki-specific formatting and HTML markup, i.e. removed links, attachments, or other formatting-specific sequences irrelevant to the actual content.

#### Contributions and active days

In order to extract information on users’ contributions, i.e. edits of Wikipedia pages made by them, we used the MediaWiki API to retrieve timestamps for each edit made by a given user. The resulting data set is publicly available in the project repository (64). The timestamps of contributions were then converted into active days. Specifically, each user *i* was represented as a binary vector $u_{i}=(a_{i1},a_{i2},\ldots ,a_{iN})$, where $a_{id}=1$ if user *i* made at least one contribution, i.e. edited a Wikipedia page, within the 24-h period corresponding to day *d* and $a_{id}=0$ otherwise. *N* is the number of days between the first recorded contribution in our data set and the last. The conversion from contribution count to active days was performed because it is hard to interpret and compare the total number of contributions between users as one large contribution could be equivalent to multiple smaller ones. Additionally, the size of a contribution does not necessarily reflect the effort put into it. While being active on a given day could still mean different levels of activity for different users, it represents a certain level of engagement with the project and is substantially different from not contributing at all on a given day.

### Toxicity

The automatic detection of offensive language in online communities has been an active area of research since at least 2010 (65). Over the past decade, researchers have focused on detecting closely-related and intersecting types of offensive language such as toxicity, abusive language, and hate speech (66), see (67) for an overview of recent advancements in the field. In this paper, we use a model from the Perspective API (68) to identify toxic comments. This is a state-of-the-art toxicity detection algorithm that obtained competitive results at OffensEval-2019 competition (69) without any additional training on the contest data and is often used as a baseline system for toxicity detection (66). Perspective API is used across multiple platforms, including The New York Times, Der Spiegel, Le Monde, and El País. It uses BERT (Bidirectional Encoder Representations from Transformers) architecture (70) and is trained on comments from a variety of online sources, including Wikipedia. Each comment is labeled by 3–10 crowdsourced raters. Perspective models provide scores for several different attributes, see Supplementary Table S2 for the list of attributes and their definitions, see Supplementary Table S2 for examples of toxic comments, and see Supplementary Table S3 for the AUC (Area Under the Curve) scores for those languages and attributes that were used in this paper.

We define a toxic comment as a comment that has a score of at least 0.8 on any of the six dimensions provided by Perspective API. The 0.8 score means that on average 8 out of 10 raters would mark it as toxic. As this threshold can be considered arbitrary, we perform additional robustness checks using different toxicity thresholds. In particular, we compute activity loss not only for the threshold of 0.8 (Table 1) but for thresholds from 0.2 to 0.9. Additionally, we applied different activity filters, e.g. we separately compute an estimate only for those users who were active at least *X* days in the past 100 days where *X* varies from 0 to 50. This is done in order to ensure that the results are not exclusively driven by those users who had made few edits and then stopped contributing to the project. We perform this analysis for English Wikipedia as it is the largest edition. As shown in Supplementary Fig. S1, the estimate is typically in the range from −0.5 to −2 and significantly lower than zero for all activity thresholds and all toxicity thresholds higher than 0.3. Similarly, we have checked how the toxicity threshold affects the probability of leaving the project. As might be expected, results remain qualitatively the same for different toxicity thresholds but higher thresholds lead to more extreme results, e.g. the probability of leaving after a toxic comment with 0.9 score is even higher than after a toxic comment with toxicity score of 0.8 (Supplementary Fig. S3).

We also evaluated the robustness of our results with respect to misclassification errors. To achieve a realistic distribution of user activity, we repeatedly sampled 100,000 editors and their activity histories from the English Wikipedia data set. These sampled users were then divided into two groups: treatment and control. We investigated two distinct scenarios: one involving an equal split between the treatment and control groups and a second, more realistic, scenario where the treatment group constituted 1% of the control group.

In the treatment group, we randomly removed one active day from each user, thereby generating a true effect of one lost active day per user. We then introduced misclassification errors by generating false positives (moving users from control to treatment group) and false negatives (moving users from treatment to control group). Finally, we compared the estimated effect, as a function of the error rate, with the true effect.

We find that, generally, misclassification leads to the underestimation of the true effect, becoming more pronounced with higher error rates (Supplementary Fig. S2). The only exception is in the case of false negatives, i.e. undetected toxic comments, in the realistic scenario. Here, misclassification does not significantly bias the estimate, though it does increase its variance.

Perspective API accepts texts up to 20,480 bytes. As the majority of comments are well below this limit, we have excluded those that are larger.

### Activity loss

Users who have received at least one toxic comment constitute our treatment group. For each user in this group, we select a random toxic comment they have received. We then center user activity around the timestamp, $t_{i}^{\mathrm{tox}}$, of that toxic comment and convert the result to active days by calculating


$$\text{sign}(|\{t\in T_{i}:t\in [t_{i}^{\text{tox}}+d*24*60*60,t_{i}^{\text{tox}}+(d+1)*24*60)\}|),$$

where $T_{i}$ is the set of timestamps of all contributions made by user *i*, and *d* is a day ranging from −100 to 100. Finally, the results are averaged over all users. We repeat the procedure of selecting a random toxic comment 100 times and report average results. However, since most users received only one toxic comment, there is little variation across simulations and the average over 100 simulations is almost identical to the result of a single simulation.

We then compare these results with a control group comprised of users who did not receive any toxic comments. However, a direct comparison is complicated because users who have received a toxic comment are, on average, more active than those who have not. This is probably due to the fact that each contribution could lead to a toxic response with a certain probability. Hence, the more contributions a user makes, the higher the likelihood of receiving a toxic comment and thereby being in the treatment group.

Specifically, if each contribution can lead to a toxic comment with a probability *p*, then the probability of receiving at least one toxic comment depends on the number of contribution, *N*: $P(\mathrm{gettoxiccomment})=1-(1-p{)}^{N}(1)$.

To ensure our control group is similarly active as the treatment group, we randomly select users with a probability based on the number of their contributions using formula (1). Users selected in this manner form the control group. For these users, we then pick a nontoxic comment at random, center their activity around its timestamp, and follow the same procedure used for the treatment group.

To test for the significance of the results, we compute 95% bootstrapped confidence intervals for each estimate.

### Probability of leaving

For each toxic comment, we find the closest in time contribution that precedes that comment. We define such contributions as “contributions followed by a toxic comment” and compare the probability of leaving after such contributions with the probability of leaving after other contributions. The probability of leaving after *N* contributions is estimated as a fraction of users who have made exactly *N* contributions among users who have made at least *N* contributions. As the probability of leaving strongly depends on *N*, we make a comparison separately for each contribution number N∈[1,100]. For N>100 the number of users is too small to provide reliable estimates for comparison.

## Supplementary Material

pgad385_Supplementary_Data

## Data Availability

The data underlying this article is available in Open Science Framework at https://osf.io/2qyxj/.

## Footnotes

a
https://meta.wikimedia.org/wiki/Data˙dumps [accessed on 2023 January 20].

b
https://mirror.accum.se/mirror/wikimedia.org/

## Previous Presentation

These results were previously presented at International Conference on Computational Social Science 2023.

## Preprints

A preprint of this article is published at https://doi.org/10.48550/arXiv.2304.13568
