# Supplementary material for: Toxic comments are associated with reduced activity of volunteer editors on Wikipedia
Source: PNAS Nexus. 2023 Dec 5;2(12):pgad385. doi: 10.1093/pnasnexus/pgad385 (PMC10697426; doi:10.1093/pnasnexus/pgad385)
Supplement: pgad385_Supplementary_Data [file pgad385_supplementary_data.pdf]

Supplementary Material

Table S1: Summary statistics for the six language editions used in the study

Edition	Comments	Users with at least one comment	Users with at least one toxic comment
English	37,304,436	6,216,906	80,307
German	8,015,691	659,167	19,543
French	3,838,161	455,598	4,282
Spanish	2,964,606	525,443	5,972
Italian	2,816,537	395,257	7,666
Russian	2,408,401	326,738	1,687

Table S2: Attributes used by Perspective API

Attribute name	Description
Toxicity	A rude, disrespectful, or unreasonable comment that is likely to make people leave a discussion.
Severe toxicity	A very hateful, aggressive, disrespectful comment or otherwise very likely to make a user leave a discussion or give up on sharing their perspective. This attribute is much less sensitive to more mild forms of toxicity, such as comments that include positive uses of curse words.
Identity attack	Negative or hateful comments targeting someone because of their identity.
Insult	Insulting, inflammatory, or negative comment towards a person or a group of people.
Profanity	Swear words, curse words, or other obscene or profane language.
Threat	Describes an intention to inflict pain, injury, or violence against an individual or group.

Table S3: Performance of Perspective API models (AUC scores)

	English	German	French	Spanish	Italian	Russian
toxicity	0.97	0.94	0.94	0.94	0.95	0.91
severe toxicity	0.98	0.98	0.96	0.96	0.97	0.95
identity attack	0.97	0.94	0.96	0.95	0.96	0.94
insult	0.97	0.93	0.94	0.93	0.96	0.92
profanity	0.99	0.97	0.98	0.98	0.98	0.96
threat	0.99	0.95	0.99	0.98	0.98	0.96

Table S4: Examples of comments with different toxicity scores

Toxicity score	Text
0.07	I wouldn't trust me on this. I suggest asking [user name], who usually goes around correcting my mistakes. Also, you appear to be vastly more qualified than me. However I would have thought that geometric isomerism would be independent of the isotopic mass - the properties would be very similar (identical?).
0.14	Your home page doesn't work. There is nothing in your talk section either!
0.21	I wanted to let you know that I just tagged [[article name]] for deletion, because it seems to be vandalism or a hoax. If you feel that the article shouldn't be deleted and want more time to work on it, you can contest this deletion, but please don't remove the speedy deletion tag from the top.
0.35	What the heck did you remove the [category name] from articles? All that happened was that some user renamed it to remove a [word] from the name, but didn't set up a redirect. Now I'm going to have to go through all the articles that were in the category and reinsert it again. Could you not have done a quick search first just to make sure that the name of the category hadn't been slightly renamed? Sorry if I sound a bit grouchy but I have spent most of this week doing nothing but fixing other people's screw-ups and I'm getting fed up having to do it.
0.49	I don't know who you think you are but I do NOT appreciate hours of work being undone. I don't know what gives you the right to undo other peoples work. You claim I am editing warring yet it feels like quite the opposite for me. I contribute to wikipedia and you trash it. Yeah, I'm not edit warring, buddy.
0.55	Hope you're proud of turning Wikipedia into a vehicle for attacking the reputations of real people whose biggest error was attempting to contribute and whose second biggest error was thinking that Wikipedia policies are sane. I'm not an edit warrior, like you, so I'm going to allow others to remove the libel. Feel free to edit war with them, too, and then pat yourself on the back for "defending Wikipedia against vandalism". The war on vandalism, like the war on drugs or terror, is a pointless and transparent fascist power grab.
0.62	Given the vile judgemental filth you have been posting in the [article name] name debate, you are not only a hypocrite; but anything I have said to you is remarkably restrained. It is clear that your only motives are to troll and to push a truly perverted point-of-view.
0.72	People have bent over backwards to accomodate you, mostly out of a pure concern for fairness and justice. You have eroded all the good will left in my body. Any more crap out of you, and I will "take steps". Better listen up good, because I'm one the oldest "old hands" around here and I know how to work the system. Don't get me riled up against you, or I will force you out!!
0.83	I would like to thank you for the personal insult to my intelligence; as opposed to addressing the arguments in my comment. I know actually engaging in dialogue would have lowered you to my level. But the rest of the public doesn't care too much for experts who believe that the rest of the public is below them, though I doubt you are an expert on anything but [vulgar speech] and being a self satisfied [expletive] who has nothing better to do than insult people on wikipedia.
0.99	Ahh shut [expletive 1] up you [expletive 2] [ethnic slur]. [expletive 1] you [ethnic slur] i will find u in real life and slit your throat.

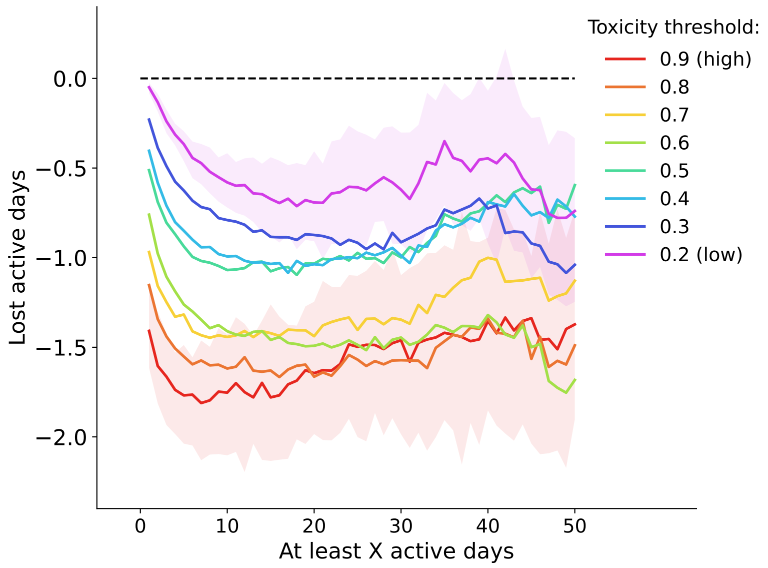

Figure S1: **Lost activity estimates as a function of toxicity threshold and activity level of users.** We find that our results are robust with respect to toxicity threshold and filtering out less active users. For visual clarity, the 95% confidence intervals (shaded regions) are shown only for 0.2 and 0.9 thresholds.

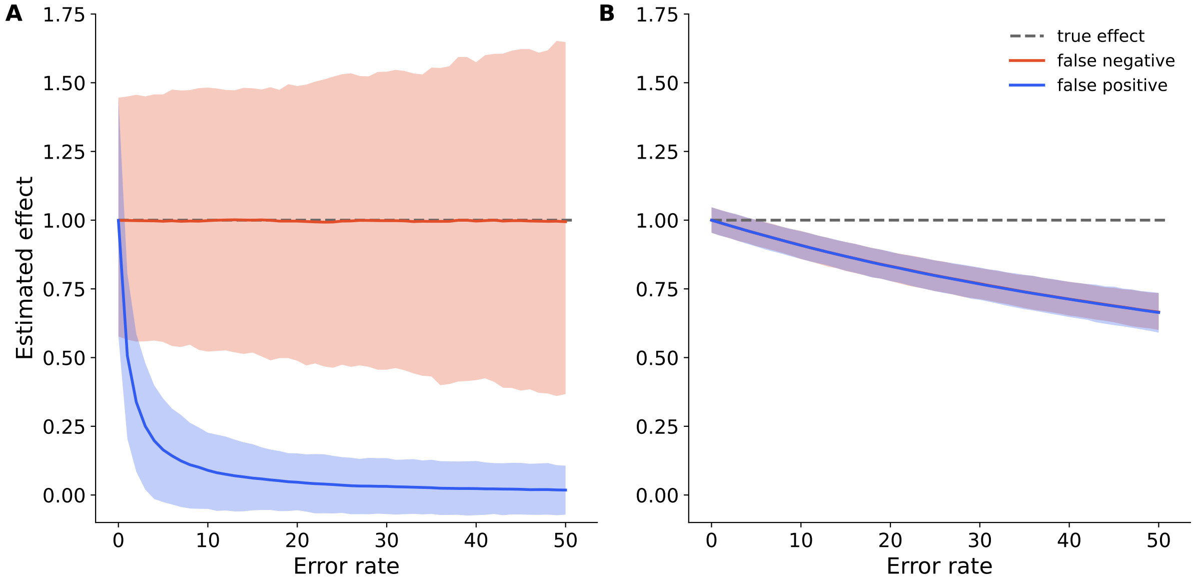

Figure S2: **Impact of classification errors on lost activity estimates.** Assuming a realistic scenario where toxic comments constitute approximately 1% of all comments (Panel A), false negatives (undetected toxic comments) do not introduce bias to the estimated effect but do induce a moderate increase in variance (red line). In contrast, false positives (misclassified non-toxic comments) lead to an underestimation of the true effect (blue line). If there is an equal number of toxic and non-toxic comments (Panel B), both false positives and false negatives have identical effects (blue and red lines coincide), consistently leading to an underestimation of the true effect, with greater error rates increasing the bias.

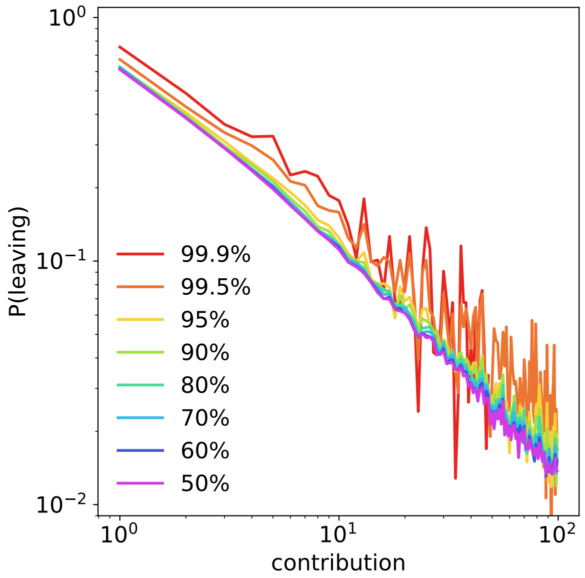

Figure S3: **Probability of leaving Wikipedia after receiving a toxic comment as a function of toxicity threshold.** As expected, the probability of leaving the project is higher when higher toxicity thresholds are used. For all thresholds, this probability is substantially higher than might be expected without a toxic comment.

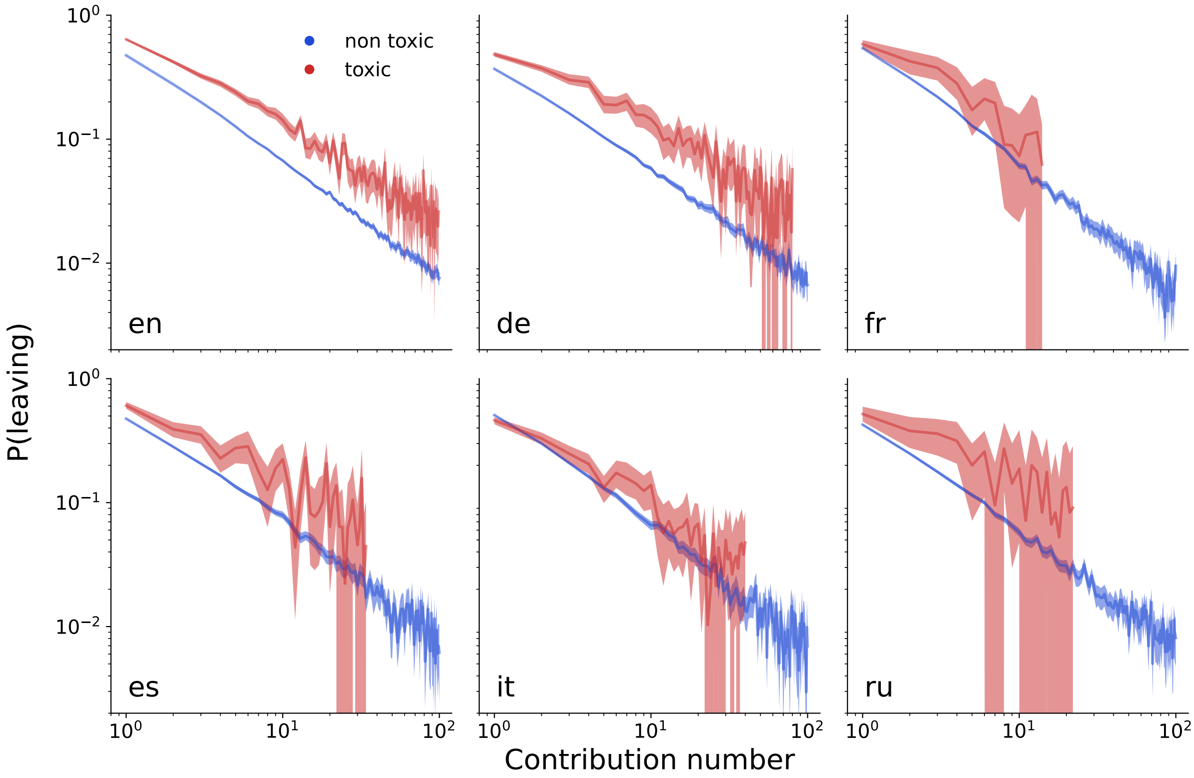

Figure S4: **Bootstrapped confidence intervals for the probability of leaving Wikipedia after receiving a toxic.** For the absolute majority of data points, the estimated probability of leaving is higher after a toxic comment than otherwise with no intersection between 95% confidence intervals making the results significant with $P < 6.25 \times 10^{-4}$. The estimates are less reliable for higher contribution numbers because of the decreasing sample size.

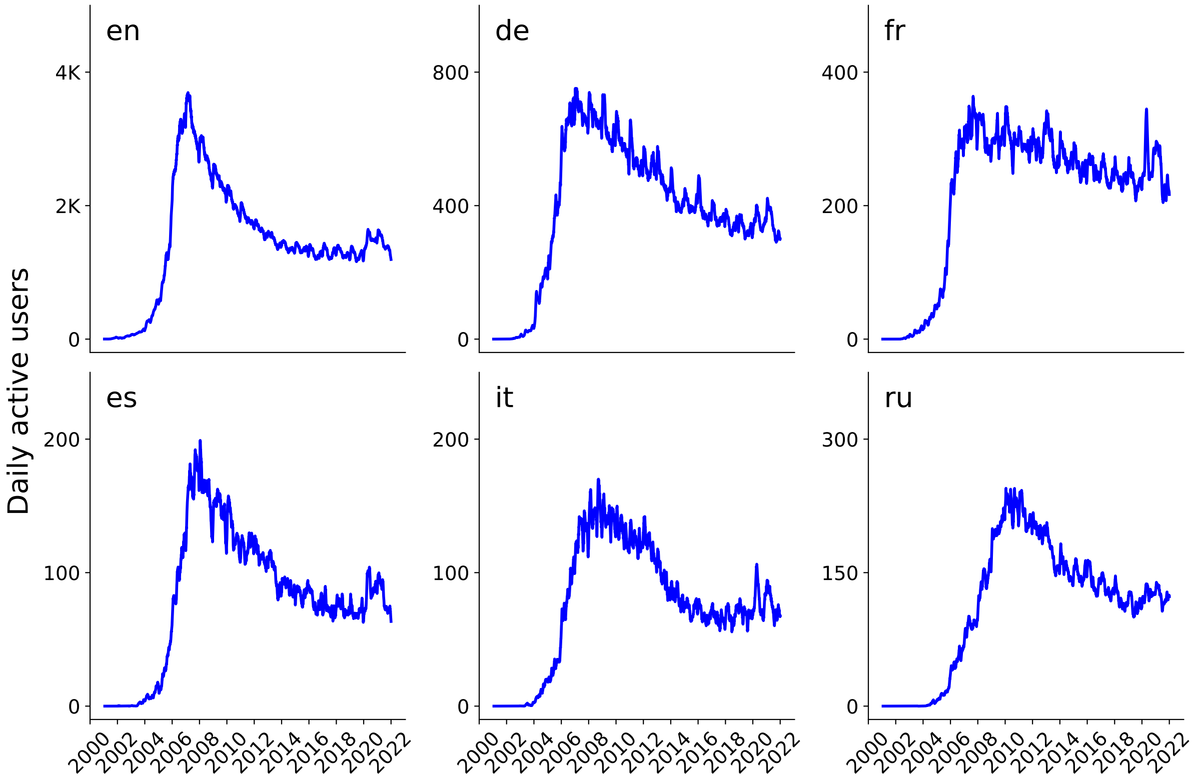

Figure S5: **The number of daily active users on Wikipedia over time.** After a period of exponential growth, the activity of Wikipedians slowed and then declined with an exception of a COVID-19-related peak in activity.

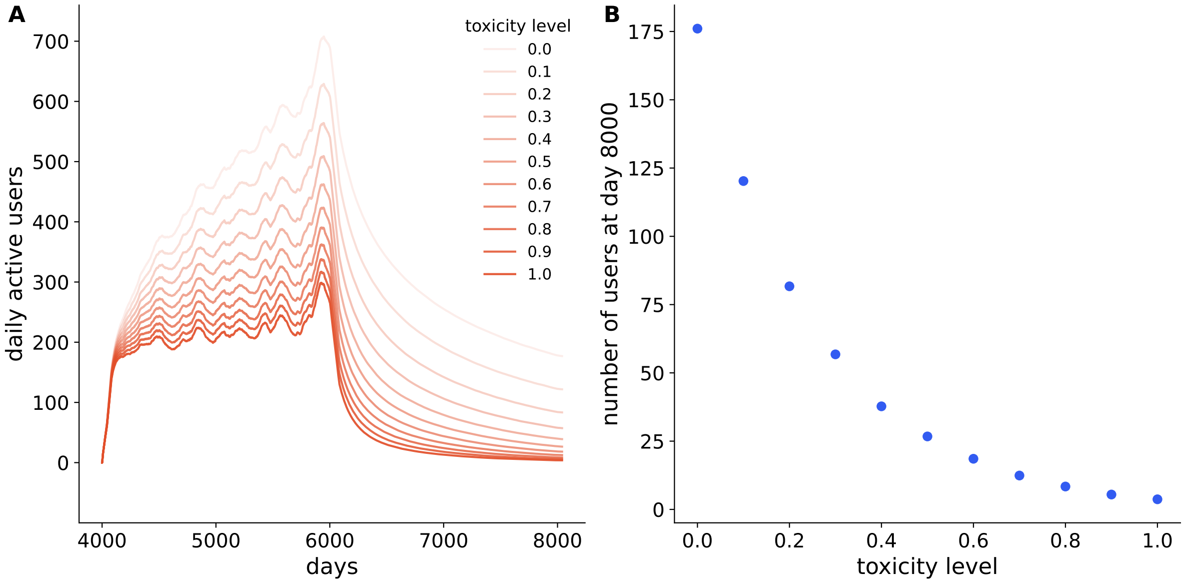

Figure S6: **The effects of toxicity levels on the number of active editors.** High levels of toxicity (darker red lines in panel A) could reduce the cohort of active editors to nearly zero, while moderate levels of toxicity could significantly decrease the number of active editors. Panel B illustrates the relationship between toxicity levels and the size of the remaining cohort over the long run.
